# Perceptions of patients on factors affecting diabetes self-management among type 2 diabetes mellitus (T2DM) patients in Fiji: A qualitative study

**DOI:** 10.1016/j.heliyon.2022.e09728

**Published:** 2022-06-15

**Authors:** Lalesh Kumar, Masoud Mohammadnezhad

**Affiliations:** School of Public Health and Primary Care, Fiji National University, Suva, Fiji

**Keywords:** Diabetes self-management, Type 2 diabetes mellitus, Patient perceptions, Barriers, Fiji

## Abstract

**Introduction:**

Optimal glycemic control can be achieved when patients are adherent to self-management behaviours such as healthy diet, physical activity, monitoring of blood glucose, reducing the risk factors, ability to solve problems and healthy coping. In light of limited studies conducted, this study aimed to explore patient's perceptions on factors affecting diabetes self-management among Type 2 Diabetes Mellitus (T2DM) patients of Labasa, Fiji.

**Materials and methods:**

A qualitative study was employed to obtain data using semi-structured interviews conducted amongst T2DM patients attending clinics in 3 randomly selected health facilities in Labasa, Fiji from 15^th^ March to 5^th^ April 2021. Non probability purposive sampling was used to recruit 30 T2DM patients. The data was collected using semi-structured open ended questionnaires. Thematic analysis was used for data analysis. This was done by closely examining the transcribed data to identify common themes such as ideas, topic and pattern that come up repeatedly, followed by reviewing themes, defining it and naming them.

**Result:**

Five themes emerged including; awareness on diabetes, perceptions towards diabetes mellitus, social support and diabetes self-management, challenges in diabetes self-management, and cultural beliefs and practices. The findings of the study demonstrated lack of knowledge and attitude towards definition of diabetes and its complications. The self-management practices amongst patients were insufficient. There was poor financial support and lack of social support among patients.

**Conclusion:**

The results of this study highlighted various factors such as poor knowledge of diabetes and its complications, inadequate family support, financial burden and strong cultural beliefs and social norms affecting diabetes self-management. This study informs the need to identify the factors affecting diabetes self-management among T2DM patients.

## 1 Introduction

Type 2 Diabetes Mellitus (T2DM) is considered a serious public health concern with huge impact on health expenditure and human life. Punthakee et al. (2018) define DM as a metabolic disorder characterized by hyperglycaemia owing to impaired insulin secretion, defective insulin action or both that is associated with long term microvascular complications and increased risk of cardiovascular diseases. According to Khan et al. (2019), diabetes affects person's quality of life and functional capacity causing morbidity and premature mortality. The study further states that more than one third of deaths attributed to diabetes occur in people under the age of 60 years. The latest estimates showed that the global prevalence of diabetes was 425 million in 2017, which is projected to increase to 629 million by 2045. This is linked to the global rise in prevalence of obesity, poor dietary choices and physical inactivity that further roots from wider societal determinants including nutrition transition (Forouhi and Wareham, 2019).

The prevalence of diabetes in Western Pacific Region (WPR) was reported to be 8.6% in 2013 or 138 million adults which is projected to rise to 11.1% in 2035. Total of 187 million deaths were attributable to diabetes in WPR in 2013 of which 44% occurred under the age of 60. In addition, the rapid rise in diabetes prevalence in WPR is possibly attributed to high rates of childhood obesity, gestational diabetes and rapid demographic and social transition (Chan et al., 2014). World Health Organization (WHO, 2021) estimates that about 121 million in the WPR had DM in 2014 and 75% of all cases diagnosed each year in WPR are in developing countries.

The prevalence of DM in Fiji was 14% in 2002 and 16% in 2011 and the prevalence of obesity was at 26% in 2002 and 32% in 2011 (Lin et al., 2015). The 2002 Stepwise Approach to Surveillance Surveys (STEPS) identified that out of 16% diabetics 50% were previously unrecognized. Diabetes is common in both i-Taukei and Fijian of Indian descent population; however, 2002 STEPS survey revealed 11.5% higher incidence of diabetes among Fijian of Indian descent population compared to the iTaukei population (MoHMS, 2020). Moreover, the 2011 STEPS survey demonstrated significant raise in blood glucose levels, blood pressure levels and obesity (Ministry of Health and Medical Services, 2018).

Poorly managed DM causes peripheral neuropathy, foot ulcers and foot sepsis resulting in lower limb amputations. Amputations are associated with increased morbidity and mortality and Fiji has the highest amputation rate in WPR (Morgan, 2015). There are also three amputations every 24 h in Fiji. Moreover, increasing diabetes prevalence leads to kidney failure which has worrying cost implications on families particularly when the need for dialysis occurs (Kumar, 2018). A survey conducted in Fiji identified that 27% of 424 diabetic eyes had evidence of maculopathy or retinopathy (Bhikoo et al., 2017).

Self–management is the key component for adequate prevention and treatment of T2DM and other Non-communicable Diseases (NCDs) as it improves health outcomes through better treatment adherence (Man et al., 2019). Adu et al. (2019) refer to diabetes self-management as day to day activities or actions a person must undertake to control or reduce the impact of disease on their health and well-being to prevent further illness. These actions involve engaging in recommended behavioural activities such as medication adherence, being active, monitoring, reducing risks, problem solving and healthy coping that are all necessary for the successful management of diabetes. Vas et al. (2017) state that DM is a chronic disease that requires regular monitoring and evaluation throughout individual's life to stay healthy. Patients limited understanding of the disease and its self-management often results in therapy failure. Knowledge and skills are required to develop positive attitude which are essential for diabetes control and consequently will reduce stress caused by illness and its treatment. Self-management can bring forth improved health care management, improve quality of life and lower associated costs (Vas et al., 2017). According to Luo et al. (2015), diabetes self-management has significant impact on improved health outcomes such as good glycaemic control, better quality of life and decreased rate of complications. According to Mikhael et al. (2018), adequate glycaemic control is essential to reduce morbidity and mortality due to DM which can be done through the prevention or delay of its complications. This can be achieved if the patients are compliant to self-management behaviours such as healthy diet, blood glucose monitoring, physical activity, healthy coping, and ability to reduce diabetes related issues.

Understanding barriers for self-care will help health care workers manage diabetes better. According to Anitha Rani and Shriraam (2019), major barriers to diabetes self-management were diet control, physical activity challenges, improper foot care, drug non-compliance and knowledge gap between patient and health care workers. Another study conducted by Tiedt and Sloan (2014) demonstrate communication barriers (distrust, misunderstanding and education methods) and organizational barriers (quality of care and access issues) as factors affecting diabetes self-management. The determinants of good diabetes self-care include social support, having care takers during illness, diabetes knowledge and microvascular complications (Ishak et al., 2017).

Given the prevalence of DM and its complications in Fiji, there had been limited studies conducted in Fiji to identify the factors affecting diabetes self–management among Fijian population. Therefore, this study aimed to explore the patient's perceptions on factors affecting diabetes self-management among T2DM patients of Labasa, Fiji.

## 2 Materials and methods

### 2.1 Study design and setting

This study applied a descriptive qualitative design that explored T2DM patient's perceptions on factors affecting diabetes self-management. It was conducted to ascertain in-depth understanding of experiences, thoughts and feelings regarding the patient's perceptions (Tenny et al., 2020). The study was conducted in three randomly selected health facilities in Macuata province of Labasa, Fiji from 15^th^ March to 05^th^ April, 2021. The random sampling was used to choose the three health facilities. This resulted in people from different locations to participate in the study as the perceptions of people may vary from different areas leading to better data saturation. These health facilities were Special Outpatient Department Labasa Hospital, Diabetic Hub Centre and Nasea health Centre that are located in Labasa medical area where special outpatient clinics for T2DM are conducted. Nasea health centre had 1300 patients, special outpatient's department Labasa hospital had 560 patients and diabetic hub centre Labasa had 295 patients. These patients either had diabetes or diabetes with other medical conditions.

### 2.2 Study sample

The target population of the study were T2DM patients in Labasa. The inclusion criteria for patients were; male and female above 18 years of age, diagnosed with T2DM for more than one year, living with or without complications, residing within Labasa medical area and attending clinics in one of the selected health facilities during the data collection period. Those who did not agree to participate in the study or mentally unable to participate in the study were excluded. Non probability purposive sampling was used to recruit T2DM patients. 20 patients from each health facility were invited to participate in the study. A total of 30 patients took part in the study.

### 2.3 Data collection tool

A semi-structured open ended questionnaire was used to guide the interviews. The questionnaire was based on the literature review and the study research questions. The sections comprised of structured questionnaire to collect demographic data and an interview guide to explore their knowledge and perception on diabetes self-management. The demographic data included age, gender, marital status, ethnicity, duration of diabetes and total members in family. The demographic data gives information on socioeconomic status of patients and probes further to ascertain factors affecting diabetes self-management. Moreover, the interview guide questioned comprised of knowledge on diabetes mellitus and questions on their experiences, living with diabetes. A bilingual translator was utilised when conducting interviews with T2DM patients. The questionnaire was examined by another researcher and discussed among four participants who met the inclusion criteria to find out if the questions were understood. The interviewer was the main researcher who had training to conduct interviews.

### 2.4 Study procedure

The study was done by providing flyers at the respective health facilities three weeks prior to the studies. Before collecting data, they were asked to sign a written consent. The information sheet and consent form were in English language, Hindi language and i-Taukei language to give freedom to participants to choose any language they desire. The in-depth interviews were conducted in the respective study setting at the patient's convenience. The interviews were conducted by the main researcher, Lalesh Kumar and the research assistant Ronika Kumar. in a room that was comfortable to study participants at each health facility. There was no relationship between the participants and the researchers that would result in bias. The participants were chosen based on the study criteria. The participants were informed of their voluntary participation and that the name and address won't be recorded. This strategy helped to avoid bias in sampling and results. The semi-structured interviews lasted for 20–30 min per patient. The data collection for in-depth interviews started with taking the participants written consent and explanation of the purpose of interview. The participant was also explained that the data will be audio recorded before the interviews begin. Written consent was taken and participants consented for data to be audio recorded. Finally, there was no offer of any kind of reimbursement for the participants to take part in the study.

### 2.5 Data management and data analysis

The raw data was transcribed and coded by the researcher, Lalesh Kumar and checked by the supervisor Dr. Masoud Mohammadnezhad. The data was transcribed verbatim on a word document by listening to the interviews. The data was analysed manually, no software were utilized for data analysis. Thematic analysis was used for data analysis. This was done by closely examining the transcribed data to identify common themes that came up repeatedly, followed by reviewing the themes, defining and naming. Thematic analysis comprised of six steps which included becoming familiar with the data, generating initial codes, generating themes, reviewing themes, defining themes and finally the write up (Braun and Clarke, 2012).

### 2.6 Study rigor

Rigor refers to the state of being careful, ensuring strict precision or being very accurate (Cypress, 2017). To increase the rigor and the precision of the study, the research instrument was examined by another researcher. The consent was obtained from research assistant to conduct the study. The venue of the in-depth interviews was checked to ensure that quiet and comfortable environment is maintained to conduct interview sessions. The allocated time set for the interview sessions were convenient to the participants. All the questionnaires were checked by principal researcher prior to the study. The data was collected until data saturation was reached. Data saturation is reached when there is no new information and the study provides maximum information on the phenomenon (Moser and Korstjens, 2018; Saunders et al., 2017).

### 2.7 Ethical consideration

Ethical approval was taken from the Fiji National University (FNU) College Human Health Research Ethics Committee (CHHREC) and also from Fiji National Research Ethics Review Committee (FNRERC). That ethical number of the study was 302.20 that were approved on the 11^th^ of December, 2020. Further approval had been taken from the Medical Superintendent Labasa Hospital and the Divisional Medical Officer Northern. Study subjects were reassured that the data collected did not include their names and all their information will be confidential. They were also informed that participating in the study is totally voluntary and they can leave the interview at any stage.

## 3 Results

### 3.1 Participant's characteristics

A total of 30 patients had face to face semi structured interviews comprised of 10 T2DM patients from each health facility. The interview among the patients was continued until the data saturation was achieved. Half of the participants were females. The age stratification showed that 40% of the participants were between 50- 59 years. Out of all participants 83% were married and 77% had less than 10 year duration of DM. The majority of participants (60%) were living in a family with more than 2 members as shown in Table 1.

**Table 1:** Demographic data of T2DM patients.

Variable	Frequency	Percentage
Gender		
Male	15	50
Female	15	50
Age (Years)		
40–49	2	7
50–59	12	40
60–69	10	33
70+	6	20
Marital Status		
Married	25	83
Single	1	4
Others	4	13
Duration of diabetes (years)		
0–10	23	77
>10	7	23
Number in family		
>2	18	60
0–2	10	33
Alone	2	7

### 3.2 Themes and sub-themes

The thematic analysis revealed 5 major themes as shown in Table 2. These were awareness on diabetes, perceptions towards diabetes, family support and diabetes self-management, challenges in diabetes self-management, and cultural beliefs and practices.

**Table 2:** Themes and sub themes for the patients.

Themes	Sub themes
Awareness on Diabetes	Concept of diabetes
	Possible diabetes complications
	Normal blood glucose levels
Perceptions towards diabetes	Importance of diabetes control
	Belief about diabetes medications
Family support and diabetes self-management practices	Diabetes self-management support
	Communication with HCWs
	Diabetes self-management practices
Challenges in diabetes self-management	Stress
	Physical activity
	Health system challenges
	Financial burden
Social norms	Obligation to religious practices
	Herbal medicine

#### 3.2.1 Theme 1: awareness on diabetes

The sub themes comprised of concept of diabetes, possible diabetes complications and normal blood glucose levels.

##### 3.2.1.1 Concept of diabetes

According to 25 participants, diabetes was defined as too much sugar in the blood that is caused by eating too much sugar and sweet things. Patients were able to relate high blood sugar with other carbohydrates which also increases high blood glucose levels. Patient's knowledge on definition of diabetes was specifically related to high blood sugar levels and eating too much sugar. Participants' knowledge about important aspect of diabetes definition such as impaired insulin secretion and defective insulin action were insufficient. The results demonstrate lack of knowledge and misconception on diabetes definition which can contribute to poor diabetes self-management leading to uncontrolled T2DM and its complications. One patient mentioned that there is too much sugar in the blood.

*“Diabetes is increased sugar level in the blood”*. P 13 (a 58 year old female)

##### 3.2.1.2 Possible diabetes complications

The majority of the patients believed that diabetes can cause complications such as blindness, stroke, heart attack, kidney failure and foot and leg amputations. However no patient knew all the complications of diabetes. The results indicate that patient's still lack knowledge on complications of diabetes. A patient mentioned about the kidney failure and the foot problems.

*“Diabetes can cause kidney failure, heart problems, sores and affects arms and legs”.* P 6 (a 57 year old female).

##### 3.2.1.3 Normal blood glucose levels

Majority patients (21) awareness on normal blood glucose was between 5 mmol/l to 10 mmol/l. Patients also stated that blood glucose above 10 can cause lot of problems. However, 9 patients stated that they were not sure of normal blood glucose levels. The results indicate that patients were either not confident in knowing normal blood glucose levels or they did not know about it. A patient voiced that normal sugar is less than 10.

*“The doctor has advised me that normal blood sugar is less than 10”.* P 23 (a 67 year old female).

#### 3.2.2 Theme 2: perceptions towards diabetes

The sub themes included importance of diabetes control and belief about diabetes medications.

##### 3.2.2.1 Importance of diabetes control

Majority patients (26) responded similarly regarding the importance of diabetes control such as being healthy, less health problems, less risk of amputations and eye problems. However, four patients stated that they were not aware of importance of controlling diabetes. The results indicate that the patients still lack in-depth understanding of importance of diabetes control such as potential cardiovascular diseases, kidney failure, hypertension and neuropathy. A participant voiced about non healing wounds, eye problems and other diseases.

*“If sugar is not controlled the injury will not heal. We can also have eye problems and other diseases”.* P 27 (a 64 year old male).

##### 3.2.2.2 Beliefs regarding diabetes medications

Half (15) of the patients stated that medication does more harm than good.

*“Don’t rely on tablets, go for diet control. Taking too much tablets will damage your kidneys”.* P 28 (a 63 year old male).

However, some patients believed that diabetes medications are good and should be taken to help control diabetes.

#### 3.2.3 Theme 3: family support and diabetes self-management practices

The subthemes consisted of diabetes management support, communication with HCWs and diabetes self-management practices.

##### 3.2.3.1 Diabetes self-management support

All (30) patients had good family support from their spouse in terms of good relationship and financial support. However, some patients had both poor social support and financial support from their children. The results demonstrate those spouses who were together had the best social support that assisted them to overcome difficulties associated with diabetes self-management. The spouse was able to assist them in mobility, house work, accompanying them to hospital and going out shopping. This support system was strengthened with social support from other family members. One respondent acknowledged the support from this wife.

*“My wife is always there for me”.* P 26 (a 60 year old male).

##### 3.2.3.2 Communication with health care workers (HCWs)

All patients stated that they had a good relationship with health care workers. The language barrier was overcome by presence of other translating nurses and doctors. There were no factors identified as barriers or challenges to effective communication or interpersonal relationship among patients and health care workers. This good communication is regarded as a facilitator to diabetes self-management. The following respondent acknowledged good relationship with their HCWs

*“Doctors and nurses are very nice”. They talk very well.* P 22 (a 65 year old male).

##### 3.2.3.3 Diabetes self-management practices

Most patient practiced diabetes self-management by reducing sugar and eating lesser sweet foods. Some patients said they had stopped smoking and reduced the amount of alcohol they used to drink. Others said they are exercising daily by doing gardening, going to farm and taking a walk. Patients also made dietary changes in terms of more vegetables, less oil, lesser fats, and reduced amount of red meat. However, some patients voiced that they could not substitute starch for lesser glycemic starch as they had plenty of it in their farm. Few participants also voiced that they had no choice to substitute diet once it was cooked as they had to adjust to family demands. Among self-management practices, none of the participants identified any stress coping mechanism. None of the patients were aware about the frequency, intensity and duration of exercise. These results suggest that the patients’ lack in-depth understanding of all aspects of diabetes self-management practices.

The respondent stressed on diet and alcohol:

*“I am taking less alcohol, eating more vegetables, eating less red meat and doing exercise everyday”.* P 18 (a 49 year old male)

#### 3.2.4 Theme 4: challenges in diabetes self-management

The sub themes comprised of stress, physical activity, health system challenges and financial burden.

##### 3.2.4.1 Stress

Patients (6) were stressed due to children who are staying away from them. One patient is worried as husband goes away for work while she has to stay alone. Another patient who stays with sickly husband has difficulty in managing herself while she has to look after her husband. Others were stressed as social welfare money was not enough to cater for the basic needs including the out of stock medications that they had to buy and dietary challenges when wife not at home. The results show that stresses were common among T2DM patients... It was also noted that patients especially elderly who are staying with their spouse or residing alone and have difficulty in coming for clinics, going out for shopping and maintaining other activities of daily living A participant voiced her stress in regards to her son and daughter in law:

*“I worry too much….crying…my daughter in law stops my son from coming to meet me. Daughter in law tells my son to pack his bags and go for good whenever my son wants to come”.* P 24 (a 72 year old female).

The following respondent was stressed with the financial constraint:

*“The social welfare money I am getting is not enough to buy things”.* P 8 (a 78 year old female).

##### 3.2.4.2 Physical activity

Physical activity was challenging as most patient were feeling weaker than before while some had developed complications such as foot ulcers and cardiovascular diseases. The patients still lack in-depth understanding of exercise which has become a challenge for them. These patients need exercise prescription through consultation with physiotherapist to improve glycaemic control and increase muscle strength. A respondent voiced difficulty in doing exercise:

*“I am having burning sensation in both my feet and it’s difficult for me to exercise”.* P 10 (a 61 year old male).

##### 3.2.4.3 Health system challenges

Common health system challenges faced by patients included medication shortages, long waiting time and missing folders. Health system barriers and challenges faced by patients comprised of unavailability of medications and missing folders. These challenges and barriers were compounded by the financial constraints and in adequate social welfare allowances to accommodate for buying medications and coming to clinics. One respondent stated the unavailability of medications:

*“Diabetes tablets are not available all the time”.* P 7 (a 41 year old male).

Another respondent mentioned about the folder issues:

"*Our folders are always missing and we also have to wait for very long time. before seen by doctor”.* P 23 (a 67 year old female).

##### 3.2.4.4 Financial burden

Majority patients had financial difficulties as they were no longer working. The financial difficulty was compounded by other family members who did not support them. Those who received social welfare allowance stated that it was never enough to cater for their basic needs. However, some patients had good support from their family especially children which relieved their financial constraints. Ten patients stated that they had poor financial support from their children as they are staying away from them after getting married. Financial burden prevalent in the study group were found to be poor financial status, poor financial support and insufficient social welfare allowance. Most patients are farmers who are unable to do farming either due to old age, diabetes complications or having poor muscle strength. Since most patients are farmers, they do not have FNPF money as well. Medical insurance coverage was also not present. The social welfare money is inadequate when they have to buy medications and other goods such as vegetables. Financial burden is aggravated by poor support especially from children which also leads to stress. These financial constraints will allow patients to compromise their diabetes self-management practices leading to uncontrolled blood glucose and risk of other comorbidities.

A respondent showed how they were abandoned by their son

*“I am staying with my husband. We were staying with our son but after three years he told us to go away. Now we are staying with our daughter. We are receiving social welfare allowance which is not enough to buy things and to come for clinics.* P 11 (a 58year old female).

#### 3.2.5 Theme 5: social norms

The sub themes included obligations to religious practices and the use of herbal medicine.

##### 3.2.5.1 Obligation to cultural practices

Some patients stated that we have to eat sweets things during religious program and festivals which we cannot always say no to. The patients have to attend religious activities and festivals throughout the year. Patients voiced that they had to take sweets and other sweet foods as they could not resist. This finding shows that patients are obliged to their social norms and find it difficult to resist the dietary offerings. This could affect their blood glucose levels.

The following patient had to take sweet things as they were attending their religious programs:

*“Sometimes I take sweets when I attend my religious programs”.* P 21 (a 53 year old male)

##### 3.2.5.2 Herbal medicine

Some patients admitted to be using herbal medicine with diabetes medications. The herbal medicine use was prevalent in the participants. Either it was taken as supplement or substituted for medications. Patients believed it has worked for others and it is working for them as well. The herbal medicine may be doing more harm than good in terms of the frequency and the dosage taken by patients. The following respondent admitted to be taking herbal medicine:

*“I try not to worry if my diabetes is high. I also take herbal medicine to control my sugar levels”.* P 30 (a 60 year old male).

## 4 Discussion

This section presents the interpretation of the study finding. The findings demonstrate that participants have insufficient knowledge on diabetes and its complications. There were lack of positive attitude towards importance of diabetes control and beliefs regarding diabetes medication. Moreover, there was better support from participants’ spouse compared to their children. The participants also faced challenges such as stress, poor compliance to physical activity, unavailability of medications and financial burden. The cultural beliefs and practices were prevalent among participants.

### 4.1 Awareness on diabetes and its complications

Evidence from studies has supported that having good knowledge and education on diabetes can result in good care and reduce diabetes complications significantly (Kassahun et al., 2016).

The findings demonstrate that participants had poor knowledge on awareness of diabetes and its complications. There were lack of knowledge on the normal blood glucose range as this would result in poor diabetes self-management at home. The findings of this study are consistent with the literature reporting lack of knowledge as one of the main barrier to diabetes self-management (Mikhael et al., 2018; Waheedi et al., 2016; Borba et al., 2019; Saunders, 2019). Another study conducted by Afaya et al. (2020) reported that more than half of the studied population had inadequate knowledge of diabetes complications. The patients' had some knowledge of diabetes complications but lack in-depth understanding of both acute and chronic complications of diabetes that may result in poor self-management as well as poor health outcomes. A study conducted by Obirikorang et al. (2016) demonstrated that participants knew the individual complications of diabetes but lack in-depth knowledge on complications which is consistent with the current study findings. The patient's perceptions on awareness of diabetes and its complications were insufficient that could be contributing to poor diabetes self-management among patients. The data suggests importance of identifying knowledge deficit among patients and properly health educating them to improve diabetes self-management.

The data suggests that patient's experiences with diabetes vary among each other. However, patients' lack in-depth understanding of importance of keeping blood glucose in control such as potential cardiovascular disease, hypertension, retinopathy and neuropathy the study managed to ascertain patient's beliefs on diabetes medications and their views on importance of diabetes control. The findings further demonstrate that patients are noncompliance to anti-diabetic medications due to their perception that medications do more harm than good that may lead to poor health outcomes. These results were concordant with the literature review conducted. A study by Hayward et al. (2015) shows that patients who were randomly assigned to tight glucose control had fewer major cardiovascular events than those assigned with standard therapy. Another study conducted by Hirakawa et al. (2014) demonstrated that consistency of glycaemic control is important to reduce the risk of vascular events and death in T2DM. Another study conducted by Bailey et al. (2012) demonstrated barriers to medications adherence such as high cost of medications, poor health status and fewer disease states. This concludes that patients are not compliance to anti-diabetic medications which could be one of the factors contributing to uncontrolled diabetes leading to adverse clinical outcomes. A study conducted by Brundisini et al. (2015) also show that poor adherence to medication regimens increases adverse outcomes for patients with T2DM. Another study conducted by Sweileh et al. (2014) show that improving knowledge of their patient about their illness might positively influence their medication adherence. The results further indicate that there need to identify these factors to improve diabetes self-management.

### 4.2 Family support and diabetes self-management practices

Poor support from children were identified, however the support from spouse was good.The findings demonstrate that patients lack socio-economic support from their children. The study was able to identify diabetes self-management support from families and health care workers and their self-management practices. The findings from this study are consistent with literature review showing positive impact of social support on diabetes self-management (Mansyur et al., 2015; Rashid et al., 2018). The participants had good communication with the HCWs which show that better exchange of information can take place during patient visit. A study conducted by Beverly et al. (2016) state that physician and patient's self-care communication is essential to achieving optimal diabetes outcomes. Majority patients had general understanding of the aspects of diabetes self-management practices but lack in-depth understanding. These findings demonstrate inadequate self-management practices among patients' that can result in poor glucose control leading to early onset of diabetes complications. These results are consistent with other studies conducted on diabetes self-management practices (Johani et al., 2015; Tewahido and Berhane, 2017; Milo and Connelly, 2019). The findings demonstrate the need to identify socio-economic factors and poor self-management practices among patients and refer them to multidisciplinary team such as social welfare services and counsellors.

### 4.3 Challenges in diabetes self-management

The study identified many challenges and barriers to diabetes self-management including stress, physical inactivity, health system challenges and financial burden. The patients' experienced stress as a result of poor support from children, difficulty in attending clinic due to diabetes complications and the ongoing financial burden from poor financial status and insufficient social welfare allowances. . Fijian culture is such that many elderly populations depend on their children for social and financial support and children abandoning their parents' lives patients to be isolated and deprived of basic support. These stresses lead to poor sleep patterns, non-compliance to medications, poor dietary patterns, indulging in smoking and alcohol and lack of interest in exercise resulting in poor diabetes self-management. Moreover stresses if not identified and addressed may lead to depression and other mental illness. Similar findings were reported by other studies (Bhattacharya, 2012; Walker et al., 2015; Houle et al., 2016). The patients' also faced difficulty in carrying out exercise regimens due to foot ulcers, foot amputations and complications such as retinopathy, and cardiovascular diseases. The data further suggests that patients with diabetes have difficulty in certain physical activity and there is need for prescribed physical activity on an individual basis. A study conducted by Yuing et al. (2019) state that aerobic and resistance training or both increase muscle strength and improve glycaemic control in people with diabetes. Another study conducted by Streckmann et al. (2014) show that balance training appears to be the most effective exercise intervention for patients with peripheral neuropathies. The prevalent health system challenges faced by patients were unavailability of medications in hospital pharmacy and missing patient folders. These challenges were compounded by the financial constraints and inadequate social welfare allowances needed to purchase medications from retail the pharmacy. These findings were consistent with other studies conducted by (Romakin &Masoud, 2019; Ong et al., 2018). The patients had poor financial status, insufficient social welfare allowances and poor financial support from children. Since most patients were farmers, there were unable to afford medical insurance cover and did not have FNPF funds. The results indicate that financial burden are also prevalent among these patients and there is need identify and refer patients to appropriate level of care. These financial constraints will further compromise patients’ diabetes self-management practices in terms of affording healthy diet, punctuality to their medical clinics and buying medications, causing uncontrolled T2DM leading to poor health outcomes. Findings of this study is similar to others studies conducted by (Vest et al., 2013; Weaver et al., 2014; Abdulrehman et al., 2016).

### 4.4 Social norms

The study identified the social norms common to Fijian of Indian descent ethnicity. These were patient's obligations to their religion such as having sweets and other sweet dishes during traditional and religious programmes that was difficult to resist. Data suggests that there are still cultural and religious practices that can affect patient's diabetes self-management. There is need to identify the religious and culturally sensitive issues and discuss with patients and their families. This data was not available during literature review and is considered a new finding. Moreover, the findings demonstrate that patients are still resorting to herbal medicine. The herbal medicine were either taken as a supplement or as a substitute to antidiabetic medications. The data suggests that health care workers should be aware of patient's cultural practices to adequately assess them and give the best health education. There has not been any study done in Fiji regarding commonly used herbal medicine for lowering blood glucose levels which may be doing more harm than good in terms of dosage, frequency and duration of herbal medicine use. A study done by Damnjanovic et al. (2015) show that more hypoglycaemia symptoms were reported in the group that used herbal dietary supplements.

### 4.5 Limitation

The limitation of the study is that the study is based in urban setting and may not fully identify social determinants of health that may be prevalent in rural communities such as strong cultural beliefs and the remoteness that may affect the accessibility, availability and affordability of the health care. Finally, there were few difficulties in getting patients as some were busy with their work schedule. It was also time consuming in requesting patients to participate in the study as they had other commitments.

## 5 Conclusion

There are many factors that have shown to affect diabetes self-management among T2DM patients. These factors are seldom addressed by HCWs during patients visit to their respective health facilities. Evidence has demonstrated the importance of multidisciplinary approach in combating these factors. In addition, patient's knowledge, attitudes, human capital and social capital also influences their diabetes self-management. Strengthening health care systems in terms of resources, infrastructure and trainings of health personells will equip health system in providing quality care the patients. Recommendations for patients include utilization of services especially when the multidisciplinary teams are out in communities. There also needs to be strong network amongst other stakeholders such as Non-Governmental Organisations (NGOs) that can provide ongoing support to the health system. The communication among primary, secondary and tertiary care is equally important in providing care that is accessible, affordable and available to patients. The patients should receive care that should enhance their knowledge and understanding and care that can help them to build social capital and strengthen their social support. Future research can be conducted in rural setting as this may obtain in-depth understanding of cultural and environmental factors affecting diabetes self-management.

## Declarations

### Author contribution statement

Lalesh Kumar: Conceived and designed the experiments; Performed the experiments; Analyzed and interpreted the data; Contributed reagents, materials, analysis tools or data; Wrote the paper.

Masoud Mohammadnezhad: Conceived and designed the experiments; Analyzed and interpreted the data; Contributed reagents, materials, analysis tools or data; Wrote the paper.

### Funding statement

This research did not receive any specific grant from funding agencies in the public, commercial, or not-for-profit sectors.

### Data availability statement

Data will be made available on request.

### Declaration of interests statement

The authors declare no conflict of interest.

### Additional information

No additional information is available for this paper.
